# 4,4′-(1,3,4-Oxadiazole-2,5-diyl)di­pyri­dinium dibromide monohydrate

**DOI:** 10.1107/S1600536810046878

**Published:** 2010-11-17

**Authors:** Meng Ting Han, Yuan Zhang

**Affiliations:** a Ordered Matter Science Research Center, College of Chemistry and Chemical, Engineering, Southeast University, Nanjing 211189, People’s Republic of China

## Abstract

In the title compound, C_12_H_10_N_4_O^+^·2Br^−^·H_2_O, the cation is approximately planar: the terminal rings make a dihedral angle of 7.91 (6)° with each other and dihedral angles of  6.02 (1) and 6.50 (8)° with the central ring. It is linked to the bromide anions and water mol­ecules by N—H⋯Br hydrogen bonds. In addition, O—H⋯Br and N—H⋯Br hydrogen bonds link these units into a three-dimensional network. C—H⋯N, C—H⋯Br and N—H⋯O inter­actions are also observed.

## Related literature

For background to the development of ferroelectric pure organic or inorganic compounds, see: Haertling *et al.* (1999 ▶); Homes *et al.* (2001 ▶). For the synthesis of compounds with potential piezoelectric and ferroelectric properties, see: Ye *et al.* (2006 ▶); Zhang *et al.* (2008 ▶). For bond-length data, see: Allen *et al.* (1987 ▶).
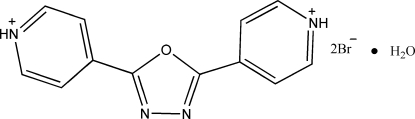


## Experimental

### 

#### Crystal data

C_12_H_10_N_4_O^+^·2Br^−^·H_2_O
*M* *_r_* = 404.08
Monoclinic, 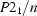

*a* = 5.2917 (11) Å
*b* = 17.531 (4) Å
*c* = 15.909 (3) Å
β = 95.42 (3)°
*V* = 1469.3 (5) Å^3^
*Z* = 4
Mo *K*α radiation
μ = 5.52 mm^−1^
*T* = 293 K
0.20 × 0.20 × 0.20 mm

#### Data collection

Rigaku Mercury2 diffractometer
Absorption correction: multi-scan (*CrystalClear*; Rigaku, 2005 ▶) *T*_min_ = 0.863, *T*_max_ = 1.000
14787 measured reflections
3351 independent reflections
2057 reflections with *I* > 2σ(*I*)
*R*_int_ = 0.124

#### Refinement

*R*[*F* ^2^ > 2σ(*F* ^2^)] = 0.061
*wR*(*F* ^2^) = 0.111
*S* = 1.06
3351 reflections
182 parameters
4 restraints
H-atom parameters constrained
Δρ_max_ = 0.50 e Å^−3^
Δρ_min_ = −0.56 e Å^−3^

### 

Data collection: *CrystalClear* (Rigaku, 2005 ▶); cell refinement: *CrystalClear*; data reduction: *CrystalClear*; program(s) used to solve structure: *SHELXS97* (Sheldrick, 2008 ▶); program(s) used to refine structure: *SHELXL97* (Sheldrick, 2008 ▶); molecular graphics: *SHELXTL* (Sheldrick, 2008 ▶); software used to prepare material for publication: *SHELXTL*.

## Supplementary Material

Crystal structure: contains datablocks I, New_Global_Publ_Block. DOI: 10.1107/S1600536810046878/jh2222sup1.cif

Structure factors: contains datablocks I. DOI: 10.1107/S1600536810046878/jh2222Isup2.hkl

Additional supplementary materials:  crystallographic information; 3D view; checkCIF report

## Figures and Tables

**Table 1:** Hydrogen-bond geometry (Å, °)

*D*—H⋯*A*	*D*—H	H⋯*A*	*D*⋯*A*	*D*—H⋯*A*
O2—H2*A*⋯Br2	1.02	2.38	3.277 (6)	147
O2—H2*A*⋯Br2	1.02	2.38	3.277 (6)	147
N1—H1*A*⋯Br1^i^	0.86	2.36	3.158 (7)	155
C12—H12*A*⋯N3^ii^	0.93	2.40	3.311 (11)	167
C10—H10*A*⋯Br1^iii^	0.93	2.75	3.595 (9)	151
N4—H4*A*⋯O2^iii^	0.86	1.78	2.608 (9)	162
C1—H1*B*⋯Br1^iv^	0.93	2.74	3.597 (9)	154
C9—H9*A*⋯Br2^iv^	0.93	2.92	3.719 (8)	145

Symmetry codes: (i) 
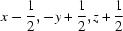
; (ii) 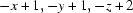; (iii) 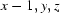; (iv) 
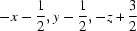
.

**Fig. 1.**
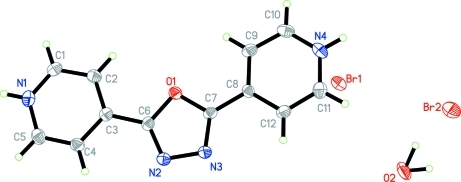
Perspective structure of the title compound, showing the atom-numbering scheme. Displacement ellipsoids are drawn at the 30% probability level.

**Fig. 2.**
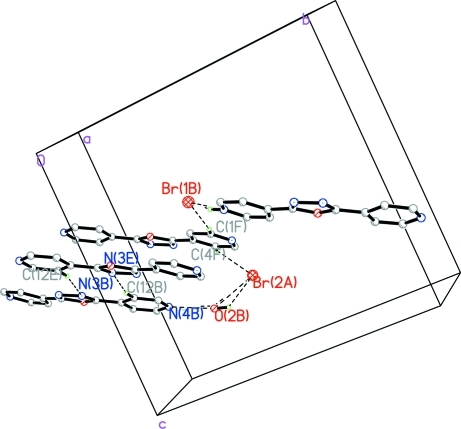
The crystal packing of the title compound viewed along the a axis showing the hydrogen bondings network. Some of the H atoms have been ommitted for clarity.

## supplementary crystallographic information

### Comment

At present, much attention in ferroelectric material field is focused on developing ferroelectric pure organic or inorganic compounds (Haertling *et al.* 1999; Homes *et al.* 2001). Recently we have reported the synthesis of a variety of compounds (Ye *et al.*, 2006; Zhang *et al.*, 2008), which have potential piezoelectric and ferroelectric. properties. In order to find more dielectric ferroelectric materials, we investigate the physical properties of the title compound(Fig. 1). The dielectric constant of the title compound as a function of temperature indicates that the permittivity is basically temperature-independent (dielectric constant equaling to 3.6 to 4.7), suggesting that this compound should be not a real ferroelectrics or there may be no distinct phase transition occurred within the measured temperature range. Similarly, below the melting point (365 K) of the compound, the dielectric constant as a function of temperature also goes smoothly, and there is no dielectric anomaly observed (dielectric constant equaling to 3.5 to 4.6).Herein, we report the synthesis and crystal structure of the title compound.

The asymmetric unit of (I) consists of one bpo dication, two bromide anions and one water molecule, linked by hydrogen bonds (Fig. 2). The bond lengths and angles are in normal ranges (Allen *et al.*, 1987). In the dication, rings A (N1/C1–C5), B (C6/N2/N3/C7/O1) and C (N4/C8–C12) are each planar. The dihedral angles between the rings are A/B = 6.02 (1), A/C =7.91 (6) and B/C =6.50 (8). As can be seen from the packing diagram (Fig. 2), molecules are connected *via* intermolecular N—H···Br and C—H···Br hydrogen bonds to form a three dimensional network. Dipole–dipole and van der Waals interactions are effective in the molecular packing.

### Experimental

A mix of 2,5-bis(4-pyridyl)-1,3,4-oxadiazole (2.24 g, 0.01 mol) and hydrobromic acid (4.05 g,0.02 mol) in methanol (20 ml) was stirred until clear. After several days, the title compound was formed and recrystallized from solution to afford colourlesss prismatic crystals suitable for X-ray analysis.

### Refinement

H atoms were positioned geometrically and refined using a riding model, with C—H = 0.93 Å and *U*_iso_(H) = 1.2_eq_(C).

### Crystal data

**Table:**

C_12_H_10_N_4_O^+^·2Br^−^·H_2_O	*F*(000) = 792
*M**_r_* = 404.08	*D*_x_ = 1.827 Mg m^−^^3^
Monoclinic, *P*2_1_/*n*	Mo *K*α radiation, λ = 0.71073 Å
Hall symbol: -P 2yn	Cell parameters from 3351 reflections
*a* = 5.2917 (11) Å	θ = 2.6–27.5°
*b* = 17.531 (4) Å	µ = 5.52 mm^−^^1^
*c* = 15.909 (3) Å	*T* = 293 K
β = 95.42 (3)°	Prism, colorless
*V* = 1469.3 (5) Å^3^	0.20 × 0.20 × 0.20 mm
*Z* = 4	

### Data collection

**Table:**

Rigaku Mercury2 diffractometer	3351 independent reflections
Radiation source: fine-focus sealed tube	2057 reflections with *I* > 2σ(*I*)
graphite	*R*_int_ = 0.124
Detector resolution: 13.6612 pixels mm^-1^	θ_max_ = 27.5°, θ_min_ = 3.5°
CCD_Profile_fitting scans	*h* = −6→6
Absorption correction: multi-scan (*CrystalClear*; Rigaku, 2005)	*k* = −22→22
*T*_min_ = 0.863, *T*_max_ = 1.000	*l* = −20→20
14787 measured reflections	

### Refinement

**Table:**

Refinement on *F*^2^	Secondary atom site location: difference Fourier map
Least-squares matrix: full	Hydrogen site location: inferred from neighbouring sites
*R*[*F*^2^ > 2σ(*F*^2^)] = 0.061	H-atom parameters constrained
*wR*(*F*^2^) = 0.111	*w* = 1/[σ^2^(*F*_o_^2^) + (0.0181*P*)^2^ + 0.7564*P*] where *P* = (*F*_o_^2^ + 2*F*_c_^2^)/3
*S* = 1.06	(Δ/σ)_max_ < 0.001
3351 reflections	Δρ_max_ = 0.50 e Å^−^^3^
182 parameters	Δρ_min_ = −0.56 e Å^−^^3^
4 restraints	Extinction correction: *SHELXL*, Fc^*^=kFc[1+0.001xFc^2^λ^3^/sin(2θ)]^-1/4^
Primary atom site location: structure-invariant direct methods	Extinction coefficient: 0.0179 (12)

### Special details

**Table:**

Geometry. All e.s.d.'s (except the e.s.d. in the dihedral angle between two l.s. planes) are estimated using the full covariance matrix. The cell e.s.d.'s are taken into account individually in the estimation of e.s.d.'s in distances, angles and torsion angles; correlations between e.s.d.'s in cell parameters are only used when they are defined by crystal symmetry. An approximate (isotropic) treatment of cell e.s.d.'s is used for estimating e.s.d.'s involving l.s. planes.
Refinement. Refinement of *F*^2^ against ALL reflections. The weighted *R*-factor *wR* and goodness of fit *S* are based on *F*^2^, conventional *R*-factors *R* are based on *F*, with *F* set to zero for negative *F*^2^. The threshold expression of *F*^2^ > σ(*F*^2^) is used only for calculating *R*-factors(gt) *etc*. and is not relevant to the choice of reflections for refinement. *R*-factors based on *F*^2^ are statistically about twice as large as those based on *F*, and *R*- factors based on ALL data will be even larger.

### Fractional atomic coordinates and isotropic or equivalent isotropic displacement parameters (Å^2^)

**Table:**

	*x*	*y*	*z*	*U*_iso_*/*U*_eq_	
Br1	0.18714 (17)	0.55914 (5)	0.63099 (6)	0.0437 (3)	
O1	0.0187 (10)	0.3400 (3)	0.9429 (3)	0.0319 (13)	
C12	0.1428 (16)	0.5397 (5)	0.8894 (5)	0.037 (2)	
H12A	0.2942	0.5478	0.9229	0.044*	
N1	0.0017 (14)	0.0806 (4)	1.0711 (5)	0.043 (2)	
H1A	−0.0375	0.0360	1.0885	0.051*	
N3	0.3649 (13)	0.4071 (4)	0.9798 (5)	0.0391 (18)	
C7	0.1437 (15)	0.4071 (5)	0.9364 (5)	0.031 (2)	
N2	0.3954 (13)	0.3359 (4)	1.0176 (5)	0.0390 (18)	
C2	−0.0995 (16)	0.1868 (4)	0.9865 (6)	0.039 (2)	
H2B	−0.2092	0.2117	0.9463	0.047*	
N4	−0.1899 (14)	0.5864 (4)	0.7948 (5)	0.0413 (19)	
H4A	−0.2572	0.6235	0.7653	0.050*	
C4	0.2829 (16)	0.1829 (5)	1.0785 (5)	0.038 (2)	
H4B	0.4331	0.2056	1.1010	0.045*	
C6	0.1883 (15)	0.2988 (4)	0.9944 (5)	0.033 (2)	
C9	−0.2029 (15)	0.4591 (5)	0.8382 (6)	0.039 (2)	
H9A	−0.2838	0.4119	0.8365	0.046*	
C1	−0.1564 (16)	0.1146 (5)	1.0141 (6)	0.040 (2)	
H1B	−0.3048	0.0903	0.9926	0.048*	
C3	0.1220 (15)	0.2216 (4)	1.0193 (5)	0.032 (2)	
C8	0.0254 (15)	0.4693 (4)	0.8867 (5)	0.031 (2)	
C11	0.0294 (18)	0.5977 (5)	0.8412 (6)	0.043 (2)	
H11A	0.1069	0.6453	0.8411	0.052*	
C10	−0.3083 (17)	0.5193 (5)	0.7925 (6)	0.042 (2)	
H10A	−0.4624	0.5133	0.7599	0.050*	
C5	0.2213 (17)	0.1121 (5)	1.1036 (6)	0.043 (2)	
H5A	0.3293	0.0856	1.1428	0.051*	
Br2	0.19209 (18)	0.82026 (5)	0.74803 (7)	0.0527 (4)	
O2	0.6881 (12)	0.7150 (3)	0.7191 (4)	0.0601 (19)	
H2D	0.6726	0.7611	0.7028	0.072*	
H2A	0.5106	0.7367	0.7060	0.072*	

### Atomic displacement parameters (Å^2^)

**Table:**

	*U*^11^	*U*^22^	*U*^33^	*U*^12^	*U*^13^	*U*^23^
Br1	0.0439 (6)	0.0500 (6)	0.0366 (6)	0.0134 (5)	0.0010 (4)	−0.0015 (4)
O1	0.033 (3)	0.037 (3)	0.026 (3)	−0.001 (3)	0.000 (3)	0.001 (3)
C12	0.032 (5)	0.042 (5)	0.036 (6)	−0.007 (4)	0.001 (4)	0.000 (4)
N1	0.048 (5)	0.040 (4)	0.041 (5)	−0.001 (4)	0.010 (4)	0.005 (4)
N3	0.037 (4)	0.035 (4)	0.043 (5)	−0.006 (4)	−0.009 (4)	0.004 (4)
C7	0.032 (5)	0.039 (5)	0.022 (5)	−0.009 (4)	0.000 (4)	−0.003 (4)
N2	0.038 (4)	0.039 (4)	0.038 (5)	−0.005 (4)	−0.007 (4)	0.004 (4)
C2	0.038 (5)	0.043 (5)	0.034 (6)	0.001 (4)	−0.001 (4)	−0.002 (4)
N4	0.042 (5)	0.049 (5)	0.033 (5)	0.017 (4)	0.004 (4)	0.006 (4)
C4	0.037 (5)	0.047 (5)	0.029 (6)	−0.003 (4)	−0.002 (4)	0.000 (4)
C6	0.036 (5)	0.036 (5)	0.026 (5)	−0.003 (4)	0.007 (4)	0.001 (4)
C9	0.035 (5)	0.048 (5)	0.033 (5)	−0.009 (5)	0.002 (4)	−0.004 (4)
C1	0.037 (5)	0.041 (5)	0.043 (6)	−0.008 (4)	0.005 (5)	−0.006 (5)
C3	0.035 (5)	0.038 (5)	0.026 (5)	−0.002 (4)	0.007 (4)	−0.003 (4)
C8	0.032 (5)	0.034 (5)	0.027 (5)	0.002 (4)	0.005 (4)	0.005 (4)
C11	0.050 (6)	0.040 (5)	0.042 (6)	0.000 (5)	0.010 (5)	0.003 (5)
C10	0.034 (5)	0.058 (6)	0.033 (6)	0.006 (5)	0.001 (4)	−0.007 (5)
C5	0.048 (6)	0.047 (6)	0.034 (6)	0.006 (5)	0.008 (5)	0.005 (4)
Br2	0.0432 (6)	0.0618 (7)	0.0532 (8)	0.0114 (5)	0.0060 (5)	0.0022 (5)
O2	0.061 (4)	0.046 (4)	0.073 (5)	0.022 (4)	0.002 (4)	0.016 (4)

### Geometric parameters (Å, °)

**Table:**

O1—C7	1.359 (9)	N4—C10	1.331 (10)
O1—C6	1.364 (9)	N4—H4A	0.8600
C12—C11	1.376 (11)	C4—C5	1.353 (11)
C12—C8	1.382 (10)	C4—C3	1.386 (11)
C12—H12A	0.9300	C4—H4B	0.9300
N1—C1	1.318 (10)	C6—C3	1.463 (11)
N1—C5	1.345 (11)	C9—C10	1.371 (11)
N1—H1A	0.8600	C9—C8	1.383 (11)
N3—C7	1.301 (10)	C9—H9A	0.9300
N3—N2	1.388 (9)	C1—H1B	0.9300
C7—C8	1.453 (11)	C11—H11A	0.9300
N2—C6	1.297 (10)	C10—H10A	0.9300
C2—C3	1.379 (11)	C5—H5A	0.9300
C2—C1	1.382 (11)	O2—H2D	0.8501
C2—H2B	0.9300	O2—H2A	1.0162
N4—C11	1.331 (11)		
			
C7—O1—C6	101.9 (6)	O1—C6—C3	119.4 (7)
C11—C12—C8	118.1 (8)	C10—C9—C8	119.2 (8)
C11—C12—H12A	120.9	C10—C9—H9A	120.4
C8—C12—H12A	120.9	C8—C9—H9A	120.4
C1—N1—C5	123.2 (8)	N1—C1—C2	119.4 (8)
C1—N1—H1A	118.4	N1—C1—H1B	120.3
C5—N1—H1A	118.4	C2—C1—H1B	120.3
C7—N3—N2	106.9 (6)	C2—C3—C4	119.2 (8)
N3—C7—O1	112.3 (7)	C2—C3—C6	121.6 (8)
N3—C7—C8	127.5 (7)	C4—C3—C6	119.3 (8)
O1—C7—C8	120.2 (7)	C12—C8—C9	120.0 (8)
C6—N2—N3	105.5 (7)	C12—C8—C7	118.9 (7)
C3—C2—C1	119.1 (9)	C9—C8—C7	121.1 (7)
C3—C2—H2B	120.4	N4—C11—C12	120.7 (8)
C1—C2—H2B	120.4	N4—C11—H11A	119.6
C11—N4—C10	122.0 (8)	C12—C11—H11A	119.6
C11—N4—H4A	119.0	N4—C10—C9	119.9 (8)
C10—N4—H4A	119.0	N4—C10—H10A	120.0
C5—C4—C3	120.0 (8)	C9—C10—H10A	120.0
C5—C4—H4B	120.0	N1—C5—C4	119.2 (9)
C3—C4—H4B	120.0	N1—C5—H5A	120.4
N2—C6—O1	113.3 (7)	C4—C5—H5A	120.4
N2—C6—C3	127.2 (8)	H2D—O2—H2A	61.4

### Hydrogen-bond geometry (Å, °)

**Table:**

*D*—H···*A*	*D*—H	H···*A*	*D*···*A*	*D*—H···*A*
O2—H2A···Br2	1.02	2.38	3.277 (6)	147
O2—H2A···Br2	1.02	2.38	3.277 (6)	147
N1—H1A···Br1^i^	0.86	2.36	3.158 (7)	155
C12—H12A···N3^ii^	0.93	2.40	3.311 (11)	167
C10—H10A···Br1^iii^	0.93	2.75	3.595 (9)	151
N4—H4A···O2^iii^	0.86	1.78	2.608 (9)	162
C1—H1B···Br1^iv^	0.93	2.74	3.597 (9)	154
C9—H9A···Br2^iv^	0.93	2.92	3.719 (8)	145

Symmetry codes: (i) *x*−1/2, −*y*+1/2, *z*+1/2; (ii) −*x*+1, −*y*+1, −*z*+2; (iii) *x*−1, *y*, *z*; (iv) −*x*−1/2, *y*−1/2, −*z*+3/2.
